# Supplementary material for: Diphthamide deficiency promotes association of eEF2 with p53 to induce p21 expression and neural crest defects
Source: Nat Commun. 2024 Apr 26;15:3301. doi: 10.1038/s41467-024-47670-1 (PMC11053169; doi:10.1038/s41467-024-47670-1)
Supplement: Supplementary file 1 — Supplementary information [file 41467_2024_47670_MOESM1_ESM.pdf]

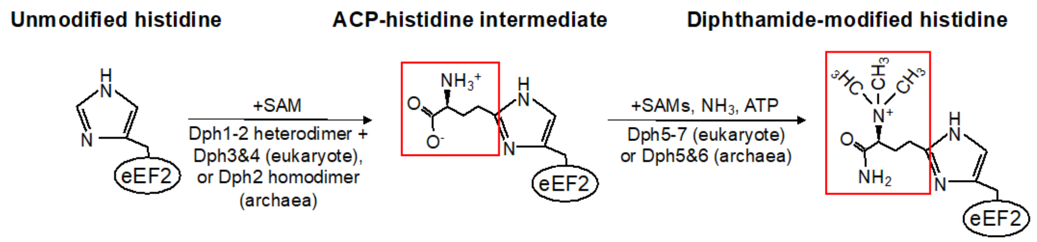

Supplementary Fig. 1. The diphthamide biosynthesis pathway in eukaryotes and archaea. Modifications of the histidine residue on eEF2 are highlighted with red boxes.

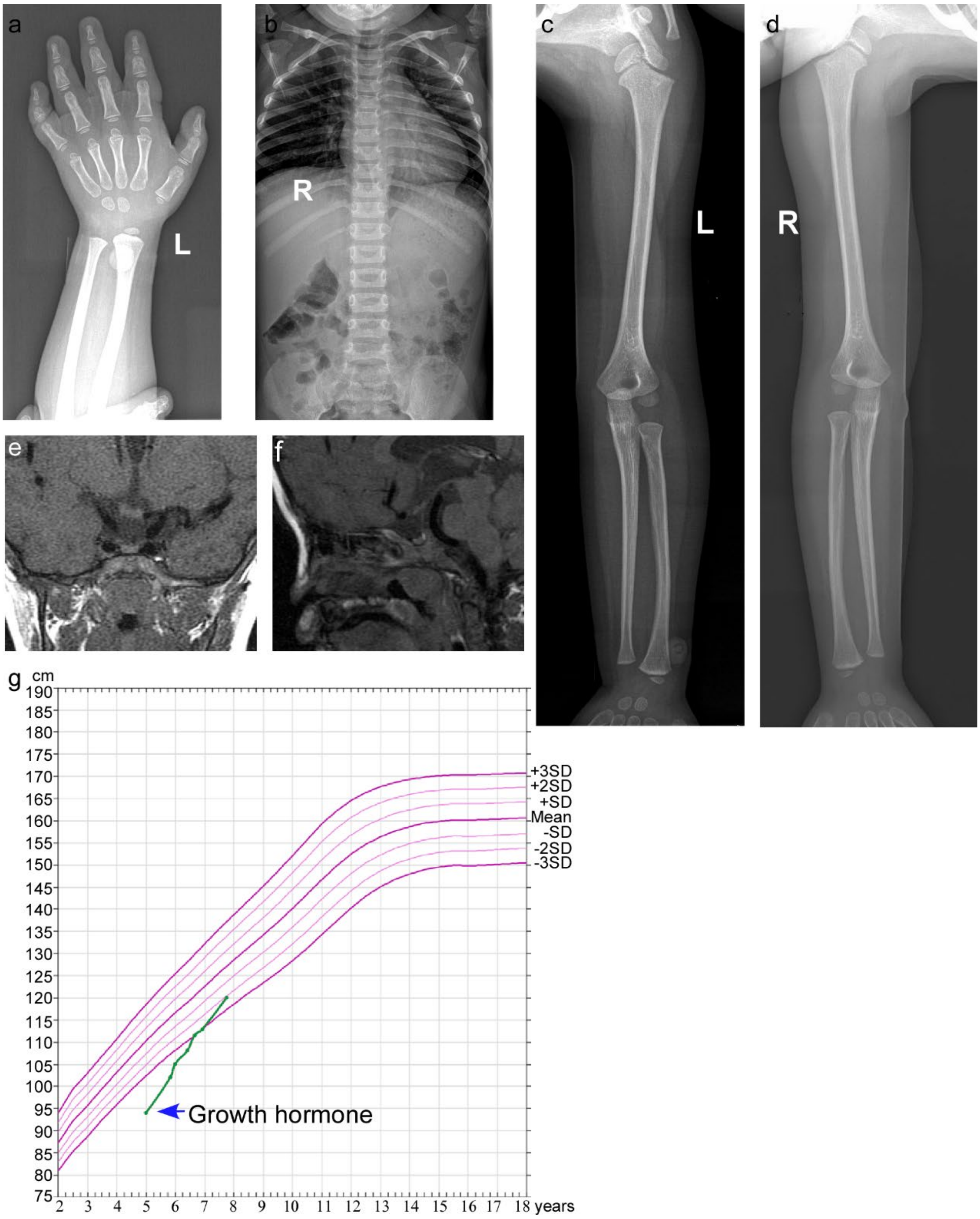

Supplementary Fig. 2. X-ray images and growth curve of the patient. a-d, X-ray images reveal delayed bone age **(a)**, but normal spine **(b)** and limb **(c, d)**. **e, f,** Coronal **(e)** and sagittal **(f)** view of T1 weighted imaging indicates normal pituitary gland. **g,** Growth curve of the proband (green), as compared with children at the same ages. SD, standard deviation. Growth hormone treatment started at age ~5 (see Supplementary Note 1).

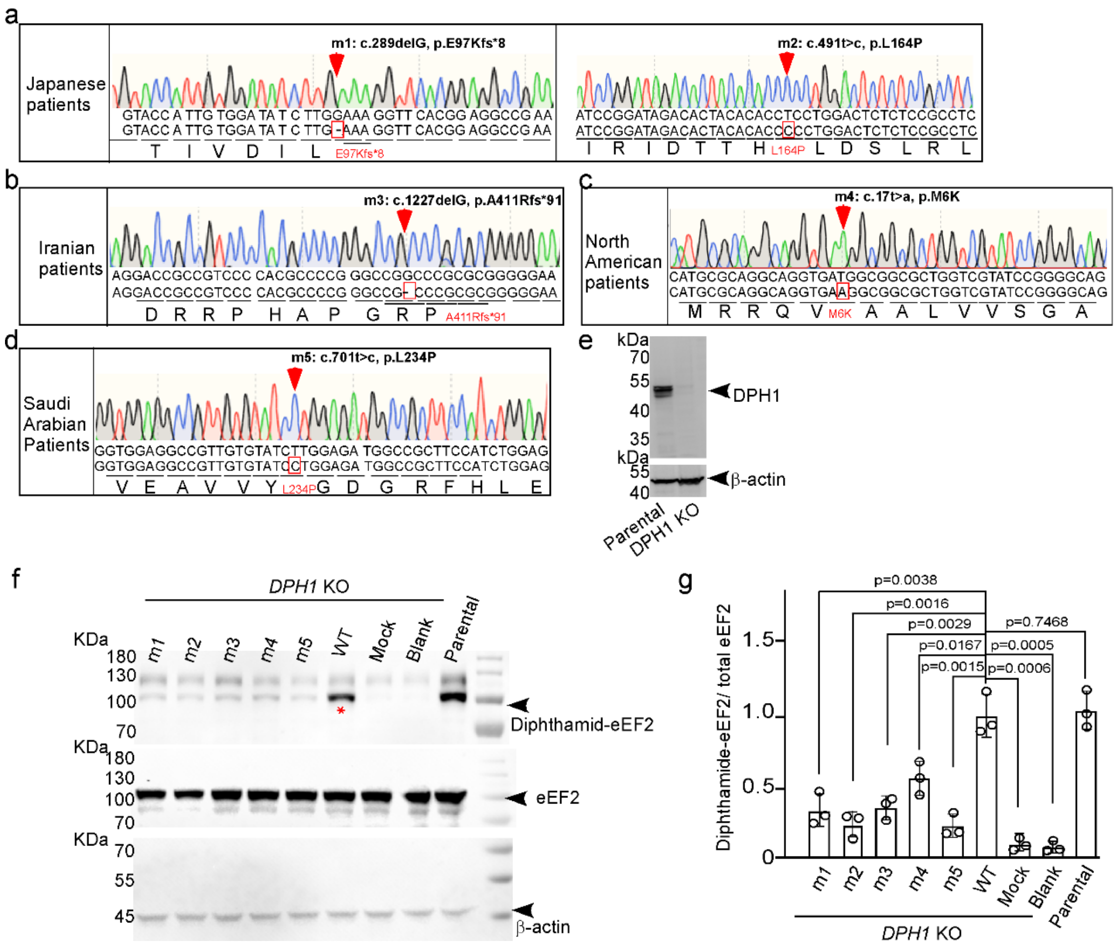

Supplementary Fig. 3. Previously identified DEDSSH-associated DPH1 mutants have reduced diphthamide biosynthesis activity. a-d, Validation of expression constructs encoding known DPH1 mutants (m1-m5) by Sanger sequencing. **e,** Western blotting confirmed the lack of DPH1 protein in *DPH1* KO HEK293T cells generated using CRISPR/Cas9 genome editing. **f,g,** *DPH1* KO HEK293T cells were transfected with the constructs expressing different DPH1 variants (0.5 μg plasmid/well in 6-well plates). Western blotting was carried out with whole-cell lysates for total and diphthamide-modified eEF2. All the mutants produced less diphthamide-modified eEF2 than wild-type (WT) DPH1 (red asterisk). A representative blot is shown in **f**, and quantification of 3 independent experiments is summarized in **g**. Values represent means ± SEM, and statistical significance was determined by unpaired *t* test with two-sided analysis. DPH1 mutants were reported in the following references: m1 and m2, Nakajima *et al.*, *J. Hum. Genet.* 2018; m3, Sekiguchi *et al.*, *J. Hum. Genet.* 2018; m4, Loucks *et al.*, *Hum. Mutat.* 2015; m5, Alazami *et al.*, *Cell Rep.* 2015.

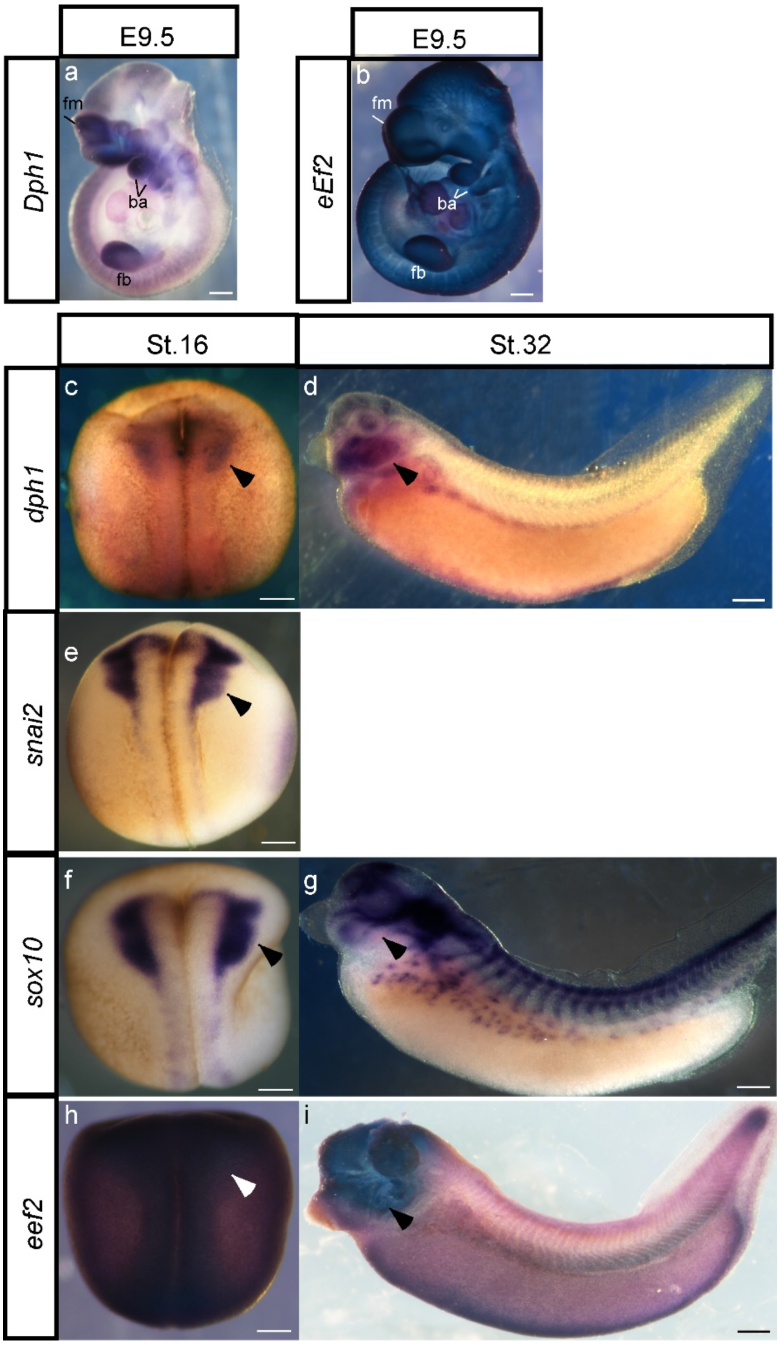

Supplementary Fig. 4. Whole-mount ISH for *Dph1* and *eEf2* mRNAs in mouse and *Xenopus* embryos. a, b, Expression of *Dph1* (a) and eEf2 (b) in E9.5 mouse embryos. fm, frontonasal mass; ba, branchial arches; fb, forelimb bud. **c-i,** Expression of *dph1* (**c, d**), *snai2* (marker for early migrating NC in **e**), *sox10* (marker for early migrating NC in **f** and differentiating NC in **g**) and *eef2* in *Xenopus* embryos at the indicated stages. Arrowheads denote early migrating (**c, e, f, h**) and differentiating (**d, g, i**) NC. Scale bar, 100 µm.

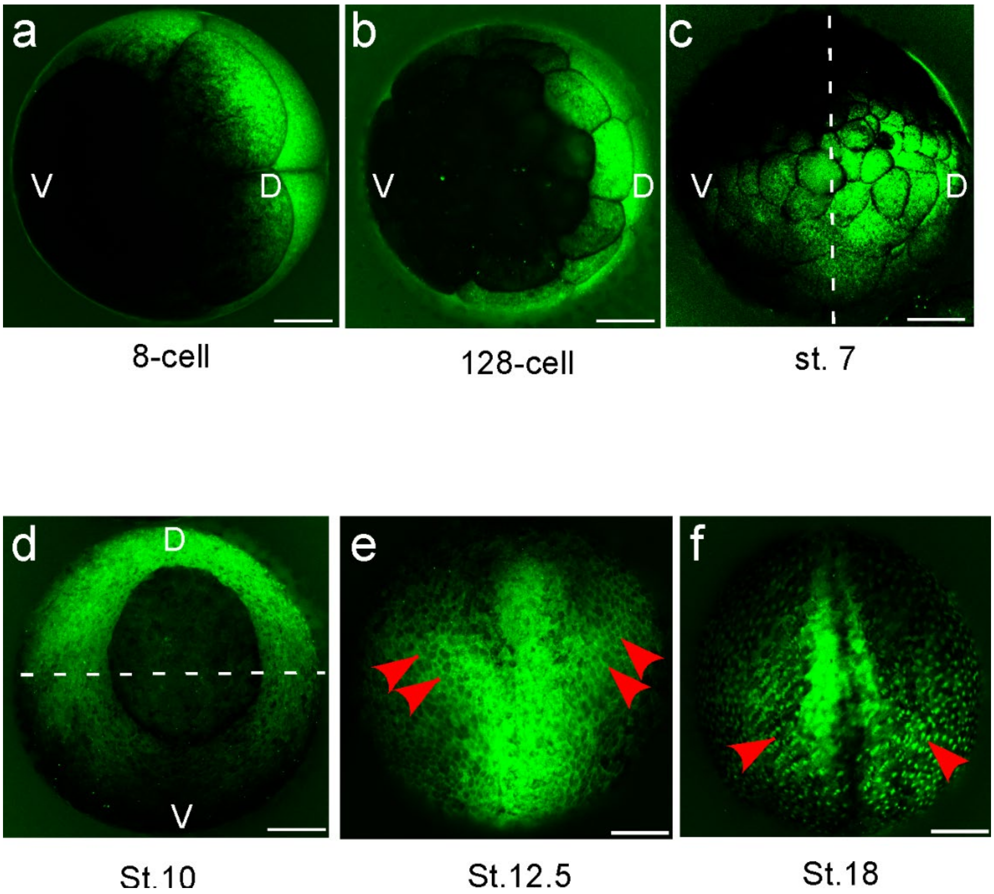

Supplementary Fig. 5. Whole-mount IHC for Dph1 protein in *Xenopus* embryos at the indicated stages. **a, b,** animal pole view. **c,** side view. **d,** vegetal pole view. **e, f,** dorsal view with anterior at the top (**e**) or bottom (**f**). Red arrowheads denote the pre-migratory (**e**) and migrating (**f**) NC. D: dorsal, V: ventral. Scale bar, 100 μm.

X. tropicalis dph1 g3 KO sequence

```
TGATGCCACGCATGGGGTGCGTATGTTATATGTTTTTGTCGACATAAAGATTGACACGTCT  wt
TTGATGCCA----TGGGGTGCGTATGTTATATGTTTTTGTCGACATAAAGATTGACACGTCT  -4    [x6]
TTGATGCC--------------------------------------------------GTCT  -50   [x2]
TTGATGCCAatcaatgggaccgtGCATGGGGTGCGTATGTTATATGTTTTTGTCGACATAAAGATTGACACGTCT  +14  [x2]
```

Supplementary Fig. 6. High indel efficiency achieved with g3-mediated genome editing in F0 *X. tropicalis* embryos. Embryos were co-injected with g3 gRNA and Cas9 protein at one-cell stage, cultured in 0.1x MBS to stage ~12.5, and genomic DNA was extracted. *dph1* gene was amplified using PCR and subcloned into a TA-cloning vector. Sequences of 10 randomly picked clones are shown in the alignment, with inserted nucleotides highlighted in red and the number of clones having the indicated sequences shown on the right. Wild-type (wt) gene sequences are included for comparison, and PAM sequence is colored in blue.

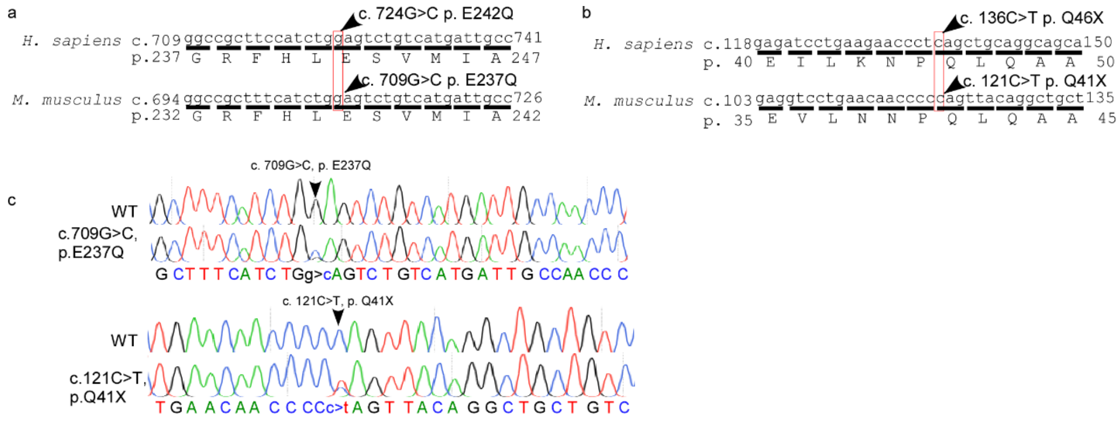

Supplementary Fig. 7. Generation of the *Dph1*$^{E237Q/Q41X}$ double knockin mice mimicking the *DPH1*$^{E242Q/Q46X}$ mutations in the proband. a, b, Sequence alignment of human and mouse Dph1 cDNAs and proteins showing the mutagenesis strategy for generating the knockin mice mimicking the mutations in the proband. The *Dph1* c. 709 G>C, p. E237Q and c. 121 C>T, p. Q41X mutations in the knockin mice correspond to the *DPH1* c. 724 G>C, p. E242Q and c.136 C>T, p. Q46X mutations in the proband, respectively. The nucleotides to be mutated (highlighted with red boxes) are conserved. **c,** Genotyping of the double knockin mice harboring the *Dph1*$^{E237Q/Q41X}$ mutations.

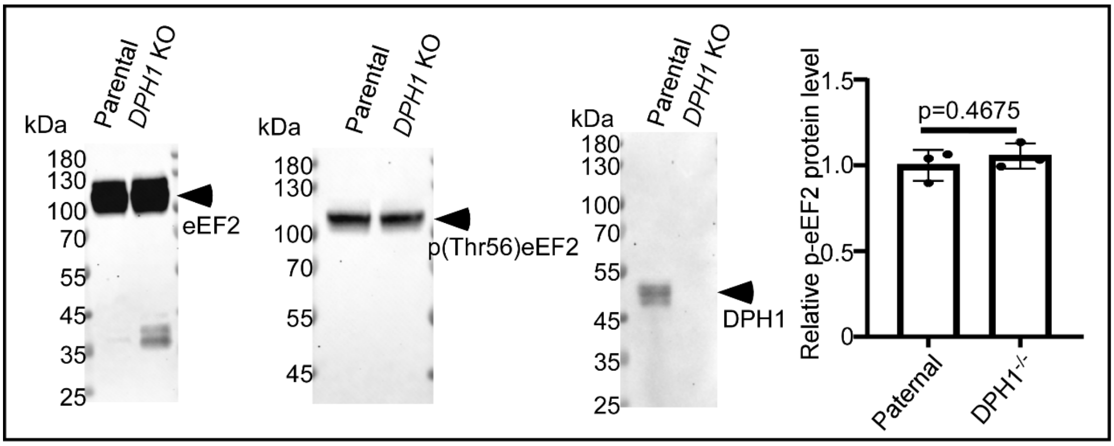

Supplementary Fig. 8. Loss of DPH1 does not affect eEF2 phosphorylation by eEF2K. Lysates of parental and *DPH1* KO U251 cells were processed for western blotting for total (left) and phospho-Thr56 (middle) eEF2 as well as DPH1 (right). Relative phospho-Thr56 eEF2 levels from 3 independent experiments are shown in the graph. ns, not significant. Values represent means ± SEM, and statistical significance was determined by unpaired *t* test with two-sided analysis.

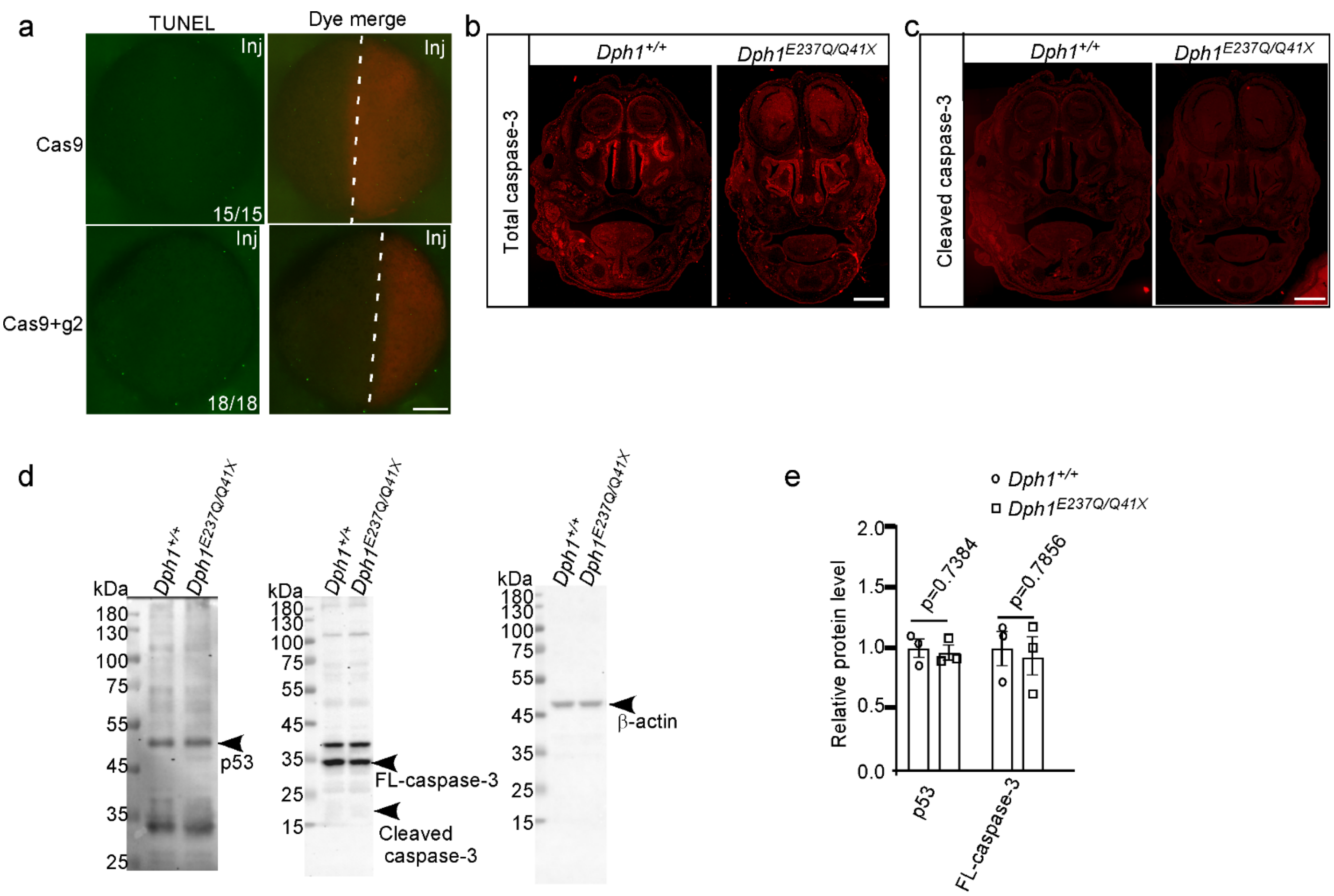

Supplementary Fig. 9. Loss of Dph1 activity does not induce apparent apoptosis in *Xenopus* or mouse embryos. a, Wild-type *X. tropicalis* embryos were injected in one blastomere at 2-cell stage with the indicated Cas9 protein and gRNA, cultured to stage ~15, and processed for TUNEL (green) assay. Injected side is denoted by co-injected Dextran 555 dye (red). The denominator in the bottom right corner of each image represents the total number of independent embryos, while the numerator indicates the number of embryos with phenotypes similar to the image (n=3 independent experiments). Scale bar, 200 µm. **b, c,** Sections of palatal shelves from E15.5 *Dph1*$^{+/+}$ and *Dph1*$^{E237Q/Q41X}$ mice were processed for IHC for total (**b**) and cleaved (**c**) caspase-3 (red), Scale bar, 500 µm. **d,** Whole-embryo lysates of E10.5 *Dph1*$^{+/+}$ and *Dph1*$^{E237Q/Q41X}$ mice were processed for western blotting for p53 and caspase-3. Representative blots are shown in **d**, and quantification of 3 independent experiments is summarized in **e**, ns, not significant. Values represent means ± SEM, and statistical significance was determined by unpaired *t* test with two-sided analysis.

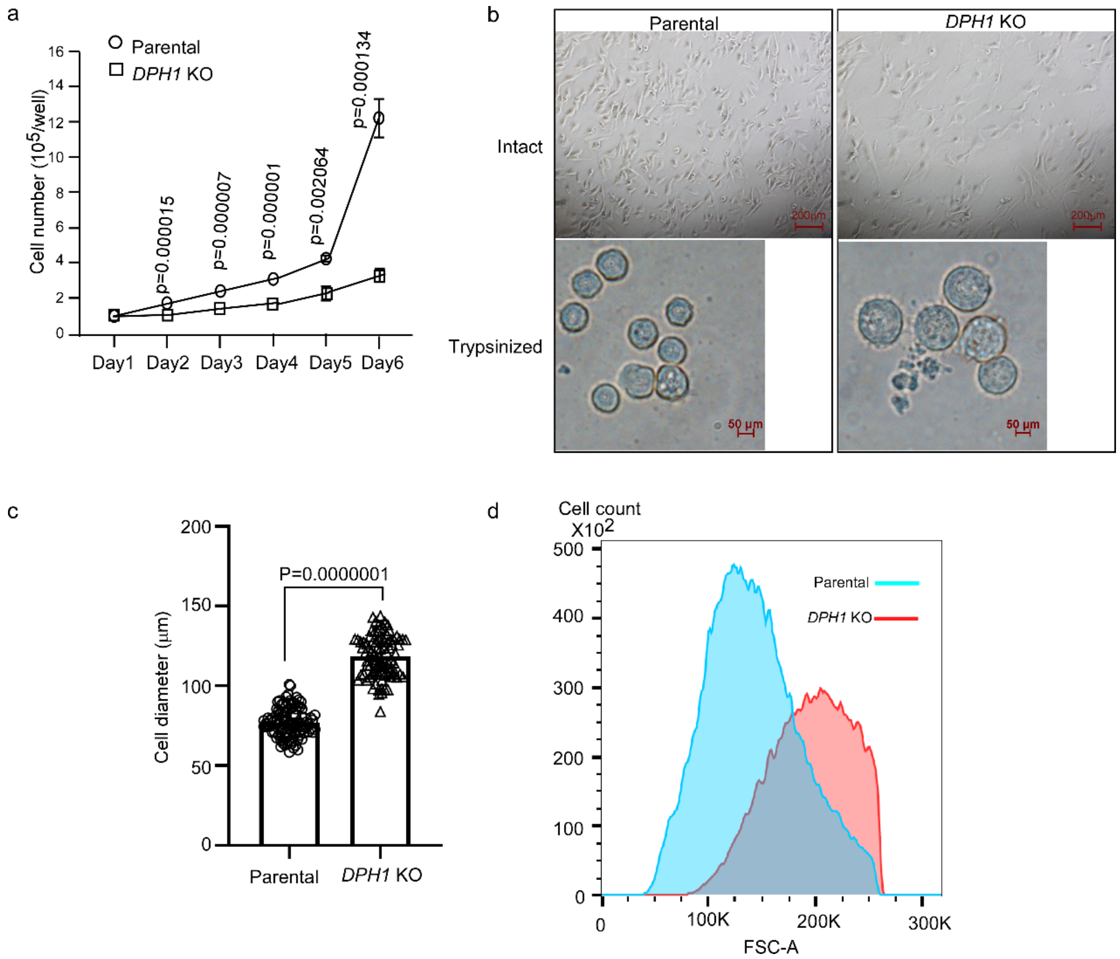

Supplementary Fig. 10. *DPH1* KO U251 cells have inhibited proliferation and enlarged size. a, Cell counting shows decreased number of *DPH1* KO U251 cells as compared with parental cells over time (n=3 independent experiments). **b-d,** *DPH1* KO U251 cells are significantly larger than parental cells. Phase-contrast images of intact (top) and trypsinized (bottom) parental and *DPH1* KO cells are shown in **b**, and diameter of 100 independent trypsinized cells are quantified and summarized in **c**. **d,** Forward scatter (FSC) measured by flow cytometry also suggests that *DPH1* KO cells are enlarged. Values represent means ± SEM, and statistical significance was determined by unpaired *t* test with two-sided analysis.

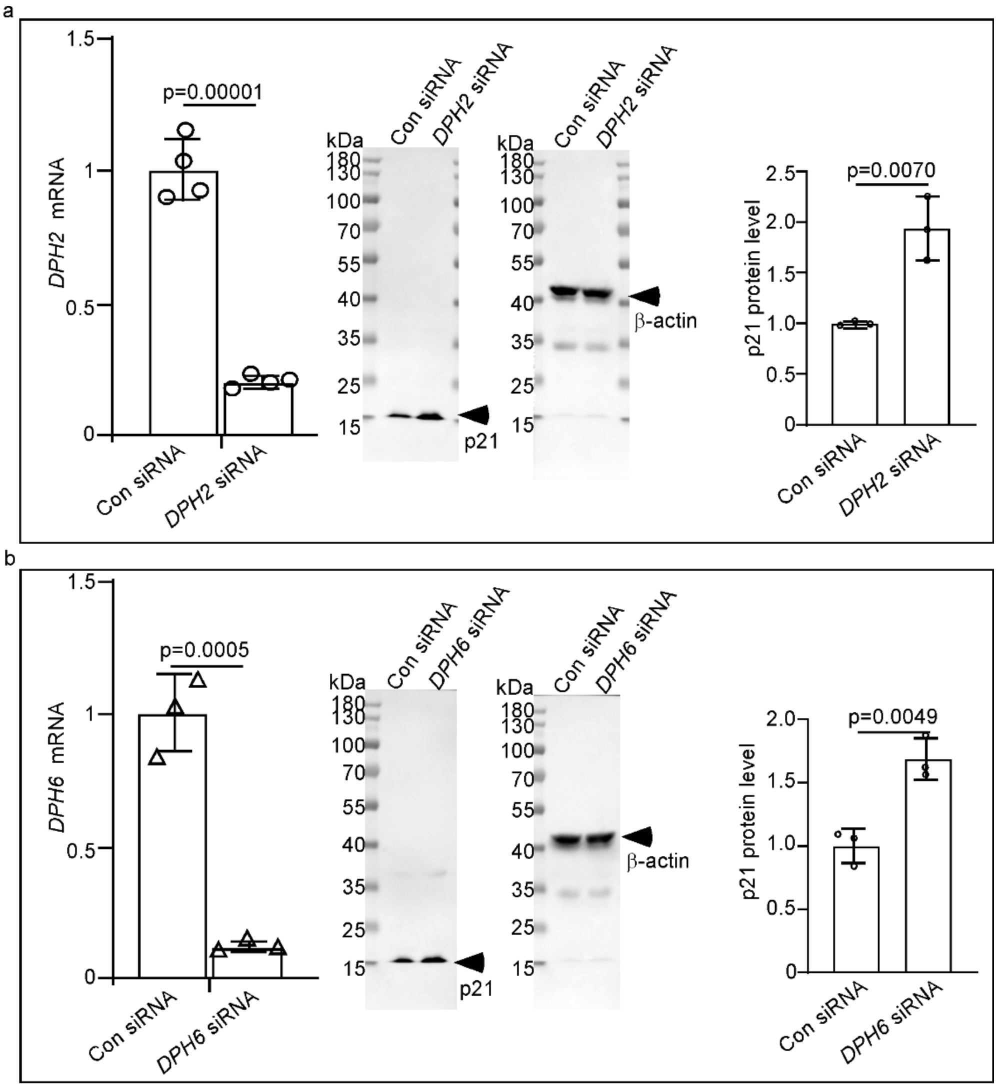

Supplementary Fig. 11. KD of either DPH2 or DPH6 results in elevated p21 levels. U251 cells were transfected with control (Con) siRNA or siRNA targeting *DPH2* (**a**) or *DPH6* (**b**). RT-qPCR for the target mRNA was carried out as a control for KD efficiency (left grafts). Cell lysates were processed for western blotting for p21, and relative p21 protein levels from 3 independent experiments are summarized in right grafts. Values represent means ± SEM, and statistical significance was determined by unpaired *t* test with two-sided analysis.

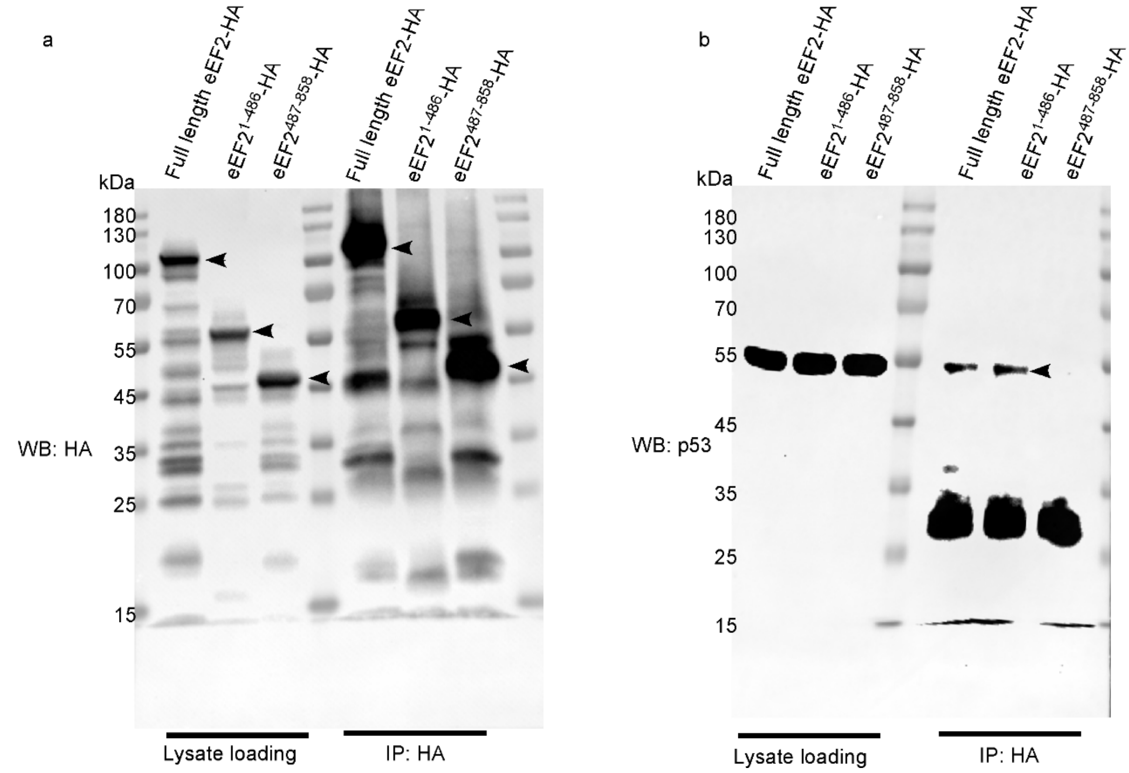

Supplementary Fig. 12. Full length and eEF2$^{1-486}$, but not eEF2$^{487-858}$, interact with p53. U251 cells were transfected with constructs encoding the indicated eEF2 variants with a C-terminal HA tag. Cell lysates were processed for IP with an anti-HA antibody, and western blotting (WB) was carried out for HA (**a**) or endogenous p53 (**b**; n=3 independent experiments). Arrowheads in **a** point to the ectopically expressed eEF2, and arrow in **b** indicates p53.

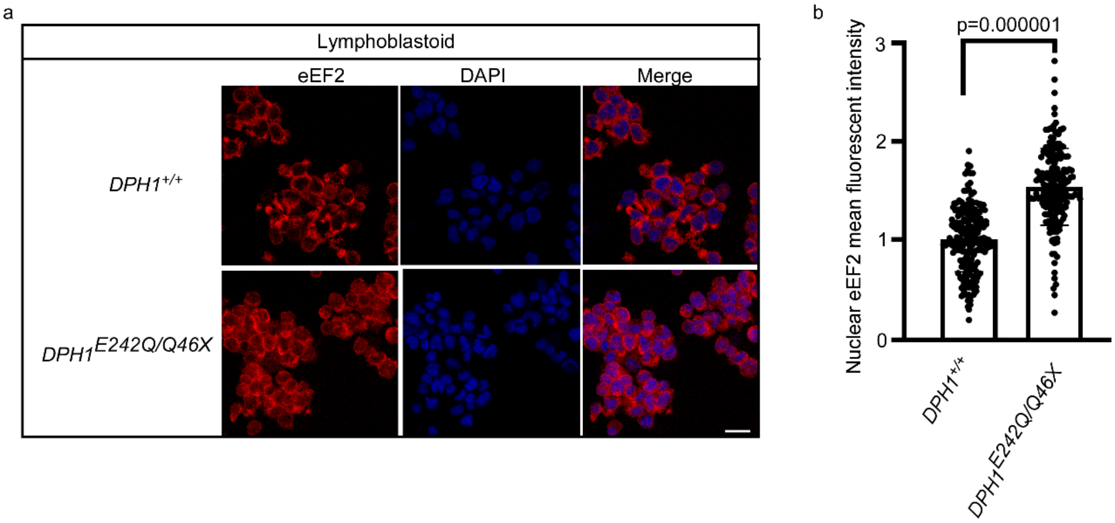

Supplementary Fig. 13. DPH1 loss of function promotes eEF2 nuclear localization. a, Immunocytochemistry for eEF2 (red) in lymphoblastoid cells from the patient (*DPH1*$^{E242Q/Q46X}$) and her sister (*DPH1*$^{+/+}$). Nuclei were stained with DAPI. **b,** Nuclear eEF2 (red fluorescence) intensity of 200 randomly picked cells of each type was quantified. Values represent means ± SEM, and statistical significance was determined by unpaired *t* test with two-sided analysis, Scale bar, 20 μm.

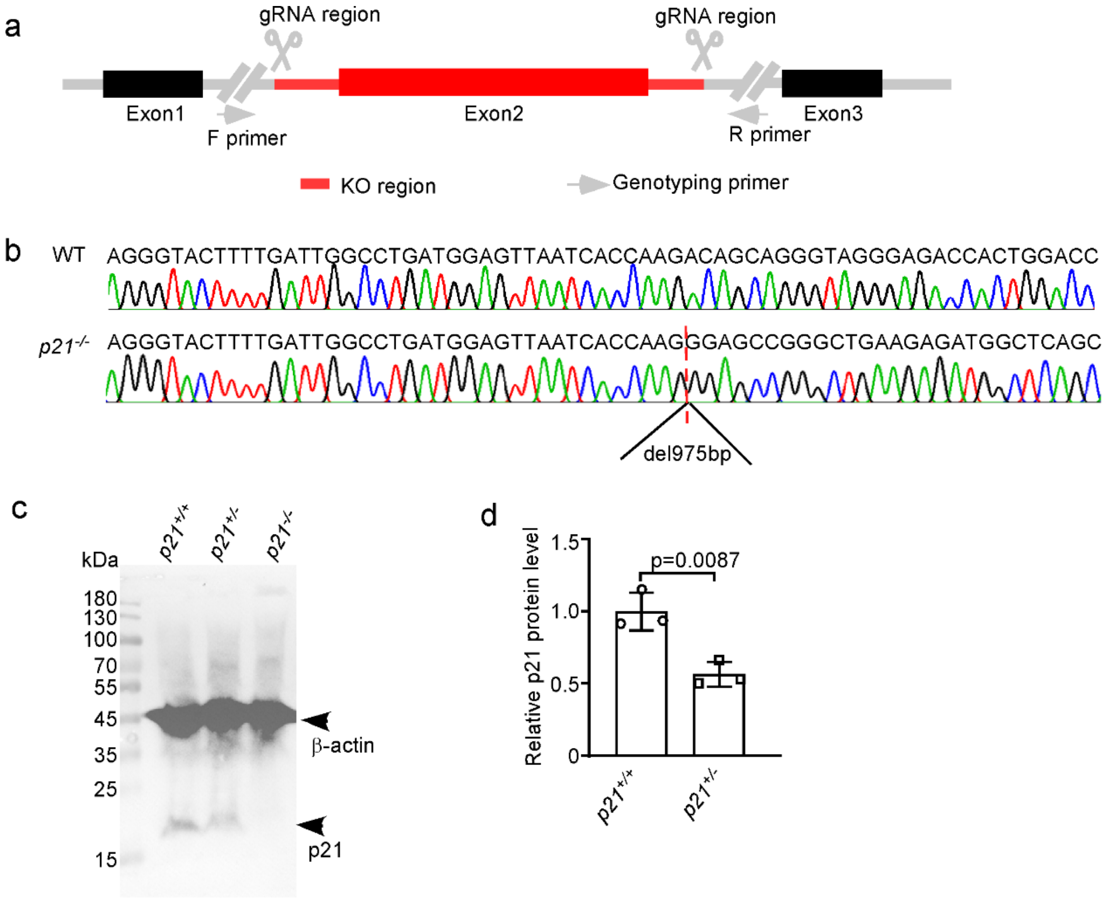

Supplementary Fig. 14. Generation of *p21* KO mice. a, Schematic diagram of the strategy for generating *p21* KO. Two guide RNAs were designed to delete a 975-bp genomic DNA fragment encompassing exon 2 of the *p21* gene, which includes the first 430 bp of the 480-bp open reading frame. Locations of the forward (F) and reverse (R) primers used for genotyping are indicated. **b,** Sanger sequencing results of the PCR products amplified from wild-type (WT) or $p21^{-/-}$ mouse genome using the genotyping primers shown in **a**. Location of the 975-bp deletion is indicated. **c,d,** Western blotting for p21 and ß-actin in $p21^{+/+}$, $p21^{+/-}$ and $p21^{-/-}$ mouse embryo lysates. A representative blot is shown in **c**, and quantification of three independent experiments is summarized in **d**. Values represent means ± SEM, and statistical significance was determined by unpaired *t* test with two-sided analysis.

Supplementary Table 1. Number of embryos born with indicated genotypes for generating the *Dph1Q41X/E237Q* compound heterozygous mice

Date genotyped	Paternal genotype	Maternal genotype	Embryonic stage	Total number	Number of *Dph1E237Q/+*	No. of *Dph1Q41X/+*	Number of *Dph1+/+*	Number of *Dph1Q41X/E237Q*
2019/11/19	Dph1Q41X/+	Dph1E237Q/+	10.5	10	1	4	4	1
2019/11/22	Dph1E237Q/+	Dph1Q41X/+	10.5	10	3	1	3	3
2019/11/29	Dph1Q41X/+	Dph1E237Q/+	10.5	8	2	2	3	1
2020/6/12	Dph1E237Q/+	Dph1Q41X/+	12.5	9	2	2	3	2
2020/6/13	Dph1E237Q/+	Dph1Q41X/+	12.5	8	1	3	2	2
2020/7/15	Dph1Q41X/+	Dph1E237Q/+	10.5	9	3	1	4	1
2020/7/16	Dph1Q41X/+	Dph1E237Q/+	10.5	6	3	1	2	0
2020/8/4	Dph1E237Q/+	Dph1Q41X/+	13.5	8	3	1	3	1
2020/8/5	Dph1Q41X/+	Dph1E237Q/+	12.5	10	4	2	3	1
2020/8/19	Dph1Q41X/+	Dph1E237Q/+	13.5	7	3	1	1	2
2020/11/26	Dph1Q41X/+	Dph1E237Q/+	13.5	8	1	3	2	2
2020/11/26	Dph1Q41X/+	Dph1E237Q/+	14.5	8	3	3	2	0
2020/11/27	Dph1E237Q/+	Dph1Q41X/+	14.5	7	1	3	2	1
2020/11/27	Dph1Q41X/+	Dph1E237Q/+	14.5	9	3	1	4	1
2021/5/26	Dph1E237Q/+	Dph1Q41X/+	14.5	7	2	2	2	1
2021/6/4	Dph1Q41X/+	Dph1E237Q/+	16.5	9	4	3	2	0
2021/6/10	Dph1Q41X/+	Dph1E237Q/+	15.5	8	1	2	3	2
2021/7/1	Dph1E237Q/+	Dph1Q41X/+	15.5	11	3	2	4	2
2021/9/7	Dph1Q41X/+	Dph1E237Q/+	10.5	8	3	2	2	1
2021/9/7	Dph1E237Q/+	Dph1Q41X/+	10.5	8	2	2	1	3
2021/9/10	Dph1Q41X/+	Dph1E237Q/+	10.5	7	4	1	2	0
2021/9/12	Dph1Q41X/+	Dph1E237Q/+	10.5	8	0	2	3	3
2021/9/12	Dph1E237Q/+	Dph1Q41X/+	10.5	8	4	2	2	0
2021/9/19	Dph1Q41X/+	Dph1E237Q/+	9.5	9	3	1	1	4
2021/10/7	Dph1E237Q/+	Dph1Q41X/+	9.5	10	2	2	2	4
2021/10/23	Dph1Q41X/+	Dph1E237Q/+	10.5	8	1	2	3	2
2021/11/19	Dph1Q41X/+	Dph1E237Q/+	10.5	11	2	4	2	3
2022/1/12	Dph1E237Q/+	Dph1Q41X/+	9.5	4	2	1	1	0
2022/1/28	Dph1Q41X/+	Dph1E237Q/+	10.5	6	1	3	1	1
2022/2/3	Dph1E237Q/+	Dph1Q41X/+	11.5	7	2	3	0	2
2022/2/3	Dph1Q41X/+	Dph1E237Q/+	10.5	4	1	2	0	1
2022/2/21	Dph1Q41X/+	Dph1E237Q/+	10.5	7	4	2	1	0
2022/2/25	Dph1E237Q/+	Dph1Q41X/+	10.5	8	1	0	5	2
2022/2/26	Dph1Q41X/+	Dph1E237Q/+	9.5	7	3	1	2	1
2022/2/26	Dph1Q41X/+	Dph1E237Q/+	9.5	8	3	1	4	0
2022/2/27	Dph1E237Q/+	Dph1Q41X/+	9.5	8	3	2	2	1
2022/3/4	Dph1Q41X/+	Dph1E237Q/+	10.5	8	2	3	1	2
2022/3/4	Dph1Q41X/+	Dph1E237Q/+	10.5	9	2	4	2	1
2023/3/10	Dph1E237Q/+	Dph1Q41X/+	16.5	5	1	2	1	1
2023/3/10	Dph1E237Q/+	Dph1Q41X/+	16.5	7	1	2	2	2
2023/3/12	Dph1E237Q/+	Dph1Q41X/+	10.5	8	3	2	1	2
2023/3/14	Dph1Q41X/+	Dph1E237Q/+	10.5	6	2	2	1	1
2023/3/14	Dph1E237Q/+	Dph1Q41X/+	10.5	7	2	2	2	1
2023/3/23	Dph1Q41X/+	Dph1E237Q/+	16.5	8	1	3	2	2
2023/3/23	Dph1E237Q/+	Dph1Q41X/+	16.5	6	3	1	1	1
2023/3/31	Dph1E237Q/+	Dph1Q41X/+	11.5	7	2	1	3	1
2023/3/31	Dph1Q41X/+	Dph1E237Q/+	11.5	8	3	1	1	3
2023/3/31	Dph1Q41X/+	Dph1E237Q/+	11.5	2	0	1	1	0
2023/6/13	Dph1Q41X/+	Dph1E237Q/+	12.5	7	1	2	2	2
2023/6/25	Dph1Q41X/+	Dph1E237Q/+	12.5	6	1	1	2	2
2023/6/26	Dph1Q41X/+	Dph1E237Q/+	12.5	6	1	2	1	2
2023/6/27	Dph1E237Q/+	Dph1Q41X/+	12.5	8	4	1	1	2
2023/6/27	Dph1E237Q/+	Dph1Q41X/+	12.5	5	2	0	2	1
Total				401	115	100	109	77

Supplementary Table 2. Number of embryos born with indicated genotypes

Date genotyped	Paternal genotype	Maternal genotype	Embryonic stage	Total No.	No. of *Dph1E237Q/+*	No. of *Dph1Q41X/+*	No. of *p21+/-*	No. of *Dph1+/+*	No. of *Dph1E237Q/+, p21+/-*	No. of *Dph1Q41X/+, p21+/-*	No. of *Dph1Q41X/E237Q*	No. of *Dph1Q41X/E237Q, p21+/-*
2022/8/26	Dph1Q41X/+, p21+/-	Dph1E237Q/+	10.5	9	0	2	2	2	0	1	0	2
2022/9/2	Dph1Q41X/+, p21+/-	Dph1E237Q/+	15.5	7	1	1	1	1	1	2	0	0
2022/9/29	Dph1Q41X/+, p21+/-	Dph1E237Q/+	15.5	11	1	1	2	1	1	1	1	3
2022/10/24	Dph1Q41X/+, p21+/-	Dph1E237Q/+	11.5	8	3	0	2	0	1	0	1	1
2022/10/26	Dph1Q41X/+, p21+/-	Dph1E237Q/+	15.5	4	0	1	1	0	0	1	1	0
2022/10/28	Dph1Q41X/+, p21+/-	Dph1E237Q/+	13.5	6	2	0	1	1	2	0	0	0
2022/10/29	Dph1Q41X/+, p21+/-	Dph1E237Q/+	11.5	9	0	0	0	3	2	0	1	3
2022/11/02	Dph1Q41X/+, p21+/-	Dph1E237Q/+	15.5	7	0	1	0	1	0	2	1	2
2022/11/04	Dph1Q41X/+, p21+/-	Dph1E237Q/+	15.5	7	3	0	0	0	2	1	0	1
2022/11/07	Dph1Q41X/+, p21+/-	Dph1E237Q/+	10.5	8	2	3	1	0	1	1	0	0
2022/11/10	Dph1Q41X/+, p21+/-	Dph1E237Q/+	10.5	6	1	1	1	2	0	0	1	0
2022/11/14	Dph1Q41X/+, p21+/-	Dph1E237Q/+	15.5	8	2	0	0	0	2	1	1	2
2022/11/14	Dph1Q41X/+, p21+/-	Dph1E237Q/+	15.5	9	2	1	1	1	2	1	0	1
2022/11/17	Dph1Q41X/+, p21+/-	Dph1E237Q/+	10.5	8	1	2	2	1	0	1	1	0
2022/11/18	Dph1Q41X/+, p21+/-	Dph1E237Q/+	10.5	5	1	1	1	0	1	1	0	0
Total				112	19	14	15	13	15	13	8	15

Supplementary Table 3. Sequencesof PCR primers(5-' to3-') used in this study

For cDNA cloning:

X. tropicalis dph1 forward:	cccctcgagatgaaagtgctgcccgaaaactc
X. tropicalis dph1 reverse:	ccctctagatcactcagtcttggtctctc
X. tropicalis eef2 forward:	cccgaattcatggtgaacttcacggtagac
X. tropicalis eef2 reverse:	gggctcgagttacagcttgtcaaggaag
Mouse *Dph1* forward:	gcggaattcatggcggcgctggttgtgtc
Mouse *Dph1* reverse:	gcgctcgagtcagggagccggcgaagtag
Mouse *eEf2* forward:	gcggaattcatggtgaacttcacagtag
Mouse *eEf2* reverse:	gcgctcgagctacagtttgtccaggaag
Human *eEF2* forward:	gcggaattcatggtgaacttcacggtag
Human *eEF2* reverse:	gcgctcgagcaatttgtccaggaagttg
Human *eEF2* (1-486aa) reverse:	gggctcgagggtgatggtgcccgtcttcac
Human *eEF2* (487-858aa) sense:	gcggaattcatgaccttcgagcacgcgcacac

For generating DNA templates to transcript in situ hybridization probes

X. tropicalis snai2 forward:	atgccacgatcttttctgg
X. tropicalis snai2 reverse:	gggtaatacgactcactatagggagaatgtgctacacaacaaccag
X. tropicalis sox10 forward:	atgagtgatgaccaaagcttg
X. tropicalis sox10 reverse:	gggtaatacgactcactatagggagatggtcttgacagtgtagtatag
X. tropicalis pax3 forward:	atgaccagcttggctggag
X. tropicalis pax3 reverse:	gggtaatacgactcactatagggagatgcaatatctggcttcagataatg
X. tropicalis zic1 forward:	atgctcctggacgccggcc
X. tropicalis zic1 reverse:	gggtaatacgactcactatagggagaaacgtaccattcgttaaaattggaag
X. tropicalis msx1 forward:	atggccccggctctgctta

X. tropicalis msx1 reverse:	gggtaatacgactcactatagggagaggagagatgatacatgctgtatcc
X. tropicalis foxd3 forward:	atgaccctgtcaggcagcag
X. tropicalis foxd3 reverse:	gggtaatacgactcactatagggagattgcgctggccatttggcta

For RT-qPCR:

Mouse *Aldh4* forward	tcaacttcaccgcgattgga
Mouse *Aldh4* reverse	acaggtgtttgaaggtgggg
Mouse *Bax* forward	gagaggtcttcttccgggtg
Mouse *Bax* reverse	agccaccctggtcttggat

Mouse *Puma* forward	tggcacgatgctatgttcct
Mouse *Puma* reverse	aggtccaccgaggagtagat
Mouse *p21* forward	cgagaacggtggaactttgac
Mouse *p21* reverse	agtgcaagacagcgacaagg
Mouse *p53* forward	cctgtgcagttgtgggtca
Mouse *p53* reverse	cataaggtaccaccacgctgt
Mouse *Mdm2* forward	gacaggagaaagcgatacagact
Mouse *Mdm2* reverse	agcttttgccatcaggcac
Human *ALDH4* forward	cctgaagcctattgcagacc
Human *ALDH4* reverse	tgaagttgatgccacagagg
Human *BAX* forward	ggacgaactggacagtaacatgg
Human *BAX* reverse	gcaaagtagaaaagggcgacaac
Human *PUMA* forward	aagagcaaatgagccaaacg
Human *PUMA* reverse	aaacgagccccactctctg
Human *p21* forward	gaggccgggatgagttgggaggag
Human *p21* reverse	cagccggcgtttggagtggtagaa
Human *p53* forward	cccctcctggcccctgtcatcttc
Human *p53* reverse	gcagcgcctcacaacctccgtcat
Human *MDM2* forward	gtgaatctacagggacgcca
Human *MDM2* reverse	ctgatccaaccaatcacctgaa
Human *TP53TG1* forward	gcaggtctggcttaccaca
Human *TP53TG1* reverse	gtgtaagtgttcgcctggtg
Human *GAPDH* forward	agggctgcttttaactctggt
Human *GAPDH* reverse	ccccacttgattttggaggga
Human *DPH1* forward	ctgaaagccgagtatcgtgtg
Human *DPH1* reverse	tgttctctggataggactttgct
Human *DPH2* forward	gacctggacggagtgtacga
Human *DPH2* reverse	tcagggaactgcaaggcaac
Human *DPH6* forward	tgatgcatttgcacctgtgg
Human *DPH6* reverse	tgtagttgtcaggcactgagg

For genotyping mouse Dph1 mutations:

E237Q forward	tggcaatagcataggtgttt
E237Q reverse	ataaggccccaggatttg
Q41X forward	ggtgggggaggaggggagaata
Q41X reverse	ttagaaggaggggcagaggaact

For genotyping mouse p21 KO

Forward	ggtgccaagtagcagcactaat
Reverse	ggacatcattccccagagtctaag

For genotyping CRISPR/Cas9-mediated DPH1 KO HEK293T and U251 cells:

Forward	ctcctcctttatggccccgcccagc
Reverse	gcgttccagcacccacctc

For generating DNA templates to transcribe gRNAs targeting X. tropicalis dph1 :

Forward-g1	taatacgactcactataGGGATGGGCGATGTGACGTAgttttagagctagaa
Forward-g2	taatacgactcactataGGGTTGAAAGTTGAAGCGAAgttttagagctagaa
Forward-g3	taatacgactcactataGGGCATCAATCGGGACTGAGgttttagagctagaa
Reverse	GAATTCTAATACGACTCACTATAgggttgacgatagagagaacGTTTTAGAGCTAGAAATAGCAAGTT

For genotyping g3-mediated X. tropicalis dph1 KO:

Forward	ccaagtccttgccaagcaaaa
Reverse	gctggtgcatttacctgaagtg

For ChIP-qPCR:

Human *P21* -2283F	agcaggctgtggctctgatt
Human *P21* -2283R	caaaatagccaccagcctcttct
Human *P21* -1391F	ctgtcctccccgaggtca
Human *P21* -1391R	acatctcaggctgctcagagtct
Human *PUMA* -152F	gcgagactgtggccttgtgt
Human *PUMA* -152R	cgttccagggtccacaaagt

For human DPH1 variants PCR validation

	agggctagctaagatgcctgg
Q46X forward	agagacatgagaggtggaggg
Q46X reverse	cagccctgtggacttctaagc
E242Q forward	aggagacgggtaggcaacag
E242Q reverse	

Supplementary Table 4. Sequences of siRNA duplexes (5-' to 3-') used

Control siRNA forward:	uucuccgaacgugucacgutt
Control siRNA reverse:	acgugacacguucggagaatt
DPH1 siRNA1 forward:	cugccuuccaacuacaacutt
DPH1 siRNA1 reverse:	aguuguaguuggaaggcagtt
DPH1 siRNA2 forward:	ccuguaccauuguggauautt
DPH1 siRNA2 reverse:	auauccacaaugguacaggtt
DPH2 siRNA forward:	gaggcgucuagaagaguautt
DPH2 siRNA reverse:	auacucuucuagacgccuctt
DPH6 siRNA forward:	ggagcuuggauacaagacatt
DPH6 siRNA reverse:	ugucuuguauccaagcucctt

Supplementary Note 1

Patient information

The patient was a girl of four years and eleven months of age, who was referred to the Endocrinology Clinic of CHCMU with remarkably short stature. Upon physical examination, her height was 93.5 cm (< -3 standard deviation (SD)), weight was 14.0 kg (-2 SD), head circumference was 51 cm (+0.9 SD) and body mass index was 16.0 kg/m^2. The patient was delivered normally with 38-week gestation and a birth weight of 2,650 g. Her parents were physically healthy and non-consanguineous. The heights of her father and mother were 163 cm and 158 cm, respectively; neither had any features observed in the patient.

The patient presented with intellectual disability and language development delay. She could not eat on her own or communicate with her parents normally, and could only speak three words. Dysmorphic features included scaphocephaly, prominent forehead, micrognathia, low-set ears, hypertelorism, downslanting palpebral fissures, epicanthal folds and depressed nasal bridge. She also had sparse eyebrows, eyelashes and hair (Fig. 1a, b). The brain magnetic resonance imaging (MRI) showed hypomyelination of bilateral frontal lobes and clear septum cyst (Fig. 1c, d). X-ray examination revealed short fifth finger, normal bone age, as well as normal spine and limbs (Extended Data Figure 1a-d). Although MRI image indicated normal pituitary morphology (Extended Data Figure 1e and 1f), growth hormone deficiency was detected, with the peak value of growth hormone after stimulation merely 4.31 ng/ml. There was also a reduced level of insulin-like growth factor-1 (IGF-1) at 28 ng/ml (normal range is 49-289 ng/ml). After recombinant human growth hormone therapy for 6 months, IGF-1 level reached 213 ng/ml, and growth was restored (Extended Data Figure 1g).

Other laboratory parameters, including blood, urine, glucose, liver function, renal function, thyroid function, blood ammonia, and lactic acid, were all normal. Serum lipid tests showed normal levels of triglyceride, cholesterol, high-density lipoprotein and low-density lipoprotein. Tandem mass spectrometry profiles and chromosomal karyotype analysis also appeared normal.
