# Supplementary material for: Diphthamide deficiency promotes association of eEF2 with p53 to induce p21 expression and neural crest defects
Source: Nat Commun. 2024 Apr 26;15:3301. doi: 10.1038/s41467-024-47670-1 (PMC11053169; doi:10.1038/s41467-024-47670-1)
Supplement: Supplementary file 3 — Reporting Summary [file 41467_2024_47670_MOESM3_ESM.pdf]

nature portfolio

Corresponding author(s): Weihong Song

Last updated by author(s): 2024-03-01

Reporting Summary

Nature Portfolio wishes to improve the reproducibility of the work that we publish. This form provides structure for consistency and transparency in reporting. For further information on Nature Portfolio policies, see our Editorial Policies and the Editorial Policy Checklist.

Please do not complete any field with "not applicable" or n/a. Refer to the help text for what text to use if an item is not relevant to your study.
For final submission: please carefully check your responses for accuracy; you will not be able to make changes later.

Statistics

For all statistical analyses, confirm that the following items are present in the figure legend, table legend, main text, or Methods section.

n/a	Confirmed	
☐	√	The exact sample size (*n*) for each experimental group/condition, given as a discrete number and unit of measurement
☐	√	A statement on whether measurements were taken from distinct samples or whether the same sample was measured repeatedly
☐	√	The statistical test(s) used AND whether they are one- or two-sided<br>*Only common tests should be described solely by name; describe more complex techniques in the Methods section.*
√	☐	A description of all covariates tested
√	☐	A description of any assumptions or corrections, such as tests of normality and adjustment for multiple comparisons
☐	√	A full description of the statistical parameters including central tendency (e.g. means) or other basic estimates (e.g. regression coefficient) AND variation (e.g. standard deviation) or associated estimates of uncertainty (e.g. confidence intervals)
☐	√	For null hypothesis testing, the test statistic (e.g. *F*, *t*, *r*) with confidence intervals, effect sizes, degrees of freedom and *P* value noted<br>*Give P values as exact values whenever suitable.*
√	☐	For Bayesian analysis, information on the choice of priors and Markov chain Monte Carlo settings
√	☐	For hierarchical and complex designs, identification of the appropriate level for tests and full reporting of outcomes
√	☐	Estimates of effect sizes (e.g. Cohen's *d*, Pearson's *r*), indicating how they were calculated

Our web collection on statistics for biologists contains articles on many of the points above.

Software and code

Policy information about availability of computer code

Data collection	NIS-Elements software for microscope
Data analysis	Canvas for Figure5 diagram drawing Quantity One for gel imaging SPSS for statistics SOAP aligner software and exome assistant program for whole exon sequence

For manuscripts utilizing custom algorithms or software that are central to the research but not yet described in published literature, software must be made available to editors and reviewers. We strongly encourage code deposition in a community repository (e.g. GitHub). See the Nature Portfolio guidelines for submitting code & software for further information.

Data

Policy information about availability of data

All manuscripts must include a data availability statement. This statement should provide the following information, where applicable:

- Accession codes, unique identifiers, or web links for publicly available datasets
- A description of any restrictions on data availability
- For clinical datasets or third party data, please ensure that the statement adheres to our policy

All the data including the original raw data are available when required

Research involving human participants, their data, or biological material

Policy information about studies with human participants or human data. See also policy information about sex, gender (identity/presentation), and sexual orientation and race, ethnicity and racism.

Reporting on sex and gender	We have only one female patient with compound heterozygous mutations in Diphthamide Biosynthesis 1 (DPH1) in this study. Based on previous case reports in DPH1 mutations, non sex and/or gender biased symptoms were observed.
Reporting on race, ethnicity, or other socially relevant groupings	Race, ethnicity and other socially relevant groupings are not considered in this study. Patients with DPH1 mutations were reported in different race globally. No race, ethnicity or other socially relevant groupings related phenotypes were observed so far.
Population characteristics	See above
Recruitment	DEDSSH is an ultra rare disease, and no more than 20 cases were reported. In this study, we report only one patient with novel compound mutations in DPH1 gene.
Ethics oversight	Patients and ethics committee of Children's Hospital of Chongqing Medical University

Note that full information on the approval of the study protocol must also be provided in the manuscript.

Field-specific reporting

Please select the one below that is the best fit for your research. If you are not sure, read the appropriate sections before making your selection.

☑ Life sciences ☐ Behavioural & social sciences ☐ Ecological, evolutionary & environmental sciences

For a reference copy of the document with all sections, see nature.com/documents/nr-reporting-summary-flat.pdf

Life sciences study design

All studies must disclose on these points even when the disclosure is negative.

Sample size	At least 3 samples for each group were chosen, and group comparing was calculated with T test
Data exclusions	No data was excluded
Replication	All replications were consistent.
Randomization	Sample or animals were allocated into WT/Parental or treatment group based on different treatment or genetic background.
Blinding	Different groups were allocated before acquisition or analyzing data

Behavioural & social sciences study design

All studies must disclose on these points even when the disclosure is negative.

Study description	
Research sample	
Sampling strategy	
Data collection	
Timing	
Data exclusions	
Non-participation	
Randomization	

Ecological, evolutionary & environmental sciences study design

All studies must disclose on these points even when the disclosure is negative.

Study description	
Research sample	
Sampling strategy	
Data collection	
Timing and spatial scale	
Data exclusions	
Reproducibility	
Randomization	
Blinding	

Did the study involve field work? ☐ Yes ☐ No

Field work, collection and transport

Field conditions	
Location	
Access & import/export	
Disturbance	

Reporting for specific materials, systems and methods

We require information from authors about some types of materials, experimental systems and methods used in many studies. Here, indicate whether each material, system or method listed is relevant to your study. If you are not sure if a list item applies to your research, read the appropriate section before selecting a response.

Materials & experimental systems

n/a	Involved in the study	
☐	√	Antibodies
☐	√	Eukaryotic cell lines
√	☐	Palaeontology and archaeology
☐	√	Animals and other organisms
☐	√	Clinical data
√	☐	Dual use research of concern
√	☐	Plants

Methods

n/a	Involved in the study	
√	☐	ChIP-seq
☐	√	Flow cytometry
☐	√	MRI-based neuroimaging

Antibodies

Antibodies used	The details of antibodies in this study were listed in Materials and Methods reagents section.
Validation	The applications of antibodies in this manuscript were according to the manufacture's instruction

Eukaryotic cell lines

Policy information about cell lines and Sex and Gender in Research

Cell line source(s)	U251 cell line was purchased from ATCC. Lymphoblastoid cell lines were derived from female patient, sibling sister and parents respectively.
Authentication	U251 cell was purchased from ATCC and passage number was less than 10 times. LCL cells were applied karyotype analysis.
Mycoplasma contamination	All cell lines were tested negative for mycoplasma contamination
Commonly misidentified lines (See ICLAC register)	None

Palaeontology and Archaeology

Specimen provenance	
Specimen deposition	
Dating methods	

☐ Tick this box to confirm that the raw and calibrated dates are available in the paper or in Supplementary Information.

Ethics oversight	

Note that full information on the approval of the study protocol must also be provided in the manuscript.

Animals and other research organisms

Policy information about studies involving animals; ARRIVE guidelines recommended for reporting animal research, and Sex and Gender in Research

Laboratory animals	Dph1E237Q/+, Dph1Q41X/+, and p21+/-mice were created in C57BL/6J by CRISPR/Cas9-mediated genome engineering. The breeding age of the mice were 2 to 8 months. Wild type and snail2-gfp *Xenopus tropicalis* were 2-3 years old.
Wild animals	This study did not involve any wild animal.
Reporting on sex	Gender-specific manifestations were not observed in human beings and animal model. So, we did not apply sex-based analysis.
Field-collected samples	All samples were taken in operation room, where the temperature is 22-25 oC, and humidity is maintained at 40%-70%. Samples were kept on ice before further processing and observed under stereo-microscope.
Ethics oversight	Methods involving live animals were carried out in accordance with the guidelines and regulations approved and enforced by the Institutional Animal Care and Use Committees at University of Delaware and Children's Hospital of Chongqing Medical University

Note that full information on the approval of the study protocol must also be provided in the manuscript.

Clinical data

Policy information about clinical studies

All manuscripts should comply with the ICMJE guidelines for publication of clinical research and a completed CONSORT checklist must be included with all submissions.

Clinical trial registration	In this study only one case was observed, and it is not a clinical trial.
Study protocol	n/a
Data collection	The DEDSSH patient made an out-of-patient visit to Children's Hospital of Chongqing Medical University and accepted some routing tests, such as MRI, X-ray, whole exon test etc. We got the informed consent from the patient and her gurdian and collected these clinical data.
Outcomes	We collect the routing physical exam data and compared with normal criteria.

Dual use research of concern

Policy information about dual use research of concern

Hazards

Could the accidental, deliberate or reckless misuse of agents or technologies generated in the work, or the application of information presented in the manuscript, pose a threat to:

No	Yes	
√	☐	Public health
√	☐	National security
√	☐	Crops and/or livestock
√	☐	Ecosystems
√	☐	Any other significant area

Experiments of concern

Does the work involve any of these experiments of concern:

No	Yes	
√	☐	Demonstrate how to render a vaccine ineffective
√	☐	Confer resistance to therapeutically useful antibiotics or antiviral agents
√	☐	Enhance the virulence of a pathogen or render a nonpathogen virulent
√	☐	Increase transmissibility of a pathogen
√	☐	Alter the host range of a pathogen
√	☐	Enable evasion of diagnostic/detection modalities
√	☐	Enable the weaponization of a biological agent or toxin
√	☐	Any other potentially harmful combination of experiments and agents

Plants

Seed stocks

Novel plant genotypes

Authentication

ChIP-seq

Data deposition

☐ Confirm that both raw and final processed data have been deposited in a public database such as GEO.

☐ Confirm that you have deposited or provided access to graph files (e.g. BED files) for the called peaks.

Data access links
May remain private before publication.

Files in database submission

Genome browser session
(e.g. UCSC)

Methodology

Replicates

Sequencing depth

Antibodies

Peak calling parameters

Data quality

Software

Flow Cytometry

Plots

Confirm that:

- [ ] The axis labels state the marker and fluorochrome used (e.g. CD4-FITC).
- [x] The axis scales are clearly visible. Include numbers along axes only for bottom left plot of group (a 'group' is an analysis of identical markers).
- [ ] All plots are contour plots with outliers or pseudocolor plots.
- [ ] A numerical value for number of cells or percentage (with statistics) is provided.

Methodology

Sample preparation	Paternal and DPH1 knock out U251cells were trypsinated and resuspended in DMEM
Instrument	BD FACSCanto plus
Software	BDFACSDiva software was used to calculate cell number and FSC-A.
Cell population abundance	At least 30000 DPH1 ko U251cells, and more than 40000 WT U251cells were measured cell size by FACSCanto plus (see extended Figure10).
Gating strategy	The FACS here was used to measure cell forward scatter (FSC) which represent cell size not for sorting . Routine MRI scan Routine magnetic

- [ ] Tick this box to confirm that a figure exemplifying the gating strategy is provided in the Supplementary Information.

Magnetic resonance imaging

Experimental design

Design type	Routine MRI scan
Design specifications	Routine magnetic resonance scan of the whole brain with the sequences including axial T1WI, T2W1, T2FLAIR and sagittal T2WI.
Behavioral performance measures	Routine scanning parameters were used and the image quality was confirmed to be diagnostically adequate by senior imaging physicians.

Imaging type(s)	Structural
Field strength	3.0T
Sequence & imaging parameters	The case underwent routine MR scan with the sequences including axial T1WI, T2W1, T2FLAIR and sagittal T2WI. The parameters of the sequence were the following: TE=3.19ms, TR=8.22ms, TI=450ms, BW=122Hz, layer thickness: 1mm, interval: 0, flip angle: 12°.
Area of acquisition	A whole brain scan was used

Diffusion MRI [ ] Used [x] Not used

Preprocessing

Preprocessing software	Images were not pre-processed
Normalization	Routine scanning did not involve a normalization step
Normalization template	Routine scanning did not involve a normalization template
Noise and artifact removal	Did not involve noise and artifact removal
Volume censoring	Did not involve volume censoring

Statistical modeling & inference

Model type and settings	No specify model was applied
Effect(s) tested	No effect tested was involved.

Specify type of analysis: [x] Whole brain [ ] ROI-based [ ] Both

Statistic type for inference

(See Eklund et al. 2016)

Only a description of the MR image was available, no statistical analysis was involved.

Correction

Did not involve any correction step

Models & analysis

n/a	Involved in the study
√	☐ Functional and/or effective connectivity
√	☐ Graph analysis
√	☐ Multivariate modeling or predictive analysis

Functional and/or effective connectivity

No functional and/or effective connectivity involved.

Graph analysis

No graph analysis involved.

Multivariate modeling and predictive analysis

No multivariate modeling and predictive analysis involved.

This checklist template is licensed under a Creative Commons Attribution 4.0 International License, which permits use, sharing, adaptation, distribution and reproduction in any medium or format, as long as you give appropriate credit to the original author(s) and the source, provide a link to the Creative Commons license, and indicate if changes were made. The images or other third party material in this article are included in the article's Creative Commons license, unless indicated otherwise in a credit line to the material. If material is not included in the article's Creative Commons license and your intended use is not permitted by statutory regulation or exceeds the permitted use, you will need to obtain permission directly from the copyright holder. To view a copy of this license, visit http://creativecommons.org/licenses/by/4.0/
